# Large-sided games do not replicate the positional worst-case scenarios of official matches in professional football

**DOI:** 10.3389/fspor.2026.1797322

**Published:** 2026-04-02

**Authors:** Lukas Herzig, Jörg Stöckert, Matthias Lochmann, Michael C. Rumpf

**Affiliations:** 1 Department of Sport Science and Sport, Friedrich-Alexander University Erlangen-Nürnberg, Erlangen, Germany; 2 1. FC Nürnberg e.V., Nürnberg, Germany; 3 Footballscience.net, Dreieich, Germany; 4 Sport Performance Research Institute New Zealand, AUT-University, Auckland, New Zealand

**Keywords:** conditioning, external load, load management, peak-match-demands, soccer (football), various-sided-games

## Abstract

The aim of this study was to determine if positional Worst-Case-Scenarios (WCS) during official games (OG) are replicated in Large-Sided Games (LSG) with a similar Area per Player (ApP) as OG. Twenty-one football players from the second team of a professional German football club were monitored during OG and LSG. WCS of 1 min (WCS1), 3 min (WCS3), and 5 min (WCS5) were calculated for OG and LSG using rolling average methods for Total Distance (TD), Medium-Speed-Running (MSR), High-Speed-Running (HSR), and Sprint Distance (SPD), in addition to low-, medium-, and high-intensity Accelerations (ACC1, ACC2, ACC3) and Decelerations (DEC1, DEC2, DEC3). LSG displayed significantly lower values (*p* < 0.05) for TD, MSR, HSR, and SPD for all positions compared to OG. ACC1-3, and DEC1-3 were also significantly (*p* < 0.05) lower in LSG compared to WCS in OG, with exceptions observed for Full Backs (FB) in ACC1 (WCS3), ACC3 (WCS1, WCS3, and WCS5), and DEC1 (WCS3); for Strikers (ST) in ACC1 and DEC1 (WCS3 and WCS5); and for Central Midfielders (CM) in ACC2 (WCS3). These findings suggest that LSG are not suitable for replicating OG' WCS1, WCS3, and WCS5. Coaches should therefore supplement with running-based drills or adjust training games to ensure that WCS demands are met.

## Introduction

Matching the physical demands of official games (OG) is fundamental to optimizing football players' athletic performance (1). Given the intermittent nature of football, relying on 90-min average values per min fails to capture the peak intensities experienced during OG and may not provide players with sufficient stimuli for those peak demands (2, 3). For this reason, research has shifted its attention toward the concept of the Worst-Case Scenario (WCS) (4), which is defined as the most intense period of play within a given time frame during an OG (4, 5). Rolling averages (6, 7) of physical variables over 30 s (8), 1–10 min (2), and 15 min (9) are used to identify WCS using 1-min (WCS1), 3-min (WCS3), and 5-min (WCS5) windows (2). Rico-Gonzalez et al. (2) reviewed differences in physical output for these time windows, indicating a clear trend of higher physical output values for shorter time windows and progressively smaller differences as WCS duration increases.

Interestingly, the external load during these WCS durations was shown to impair subsequent physical performance (10–12). Indeed, both locomotor variables, such as total distance (TD), high-speed running (HSR), and sprint distance (SPD), as well as mechanical variables, including accelerations (ACC) and decelerations (DEC), have been reported to decrease below the OG average following WCS1, WCS3, and WCS5 (10, 12). Although TD, ACC, and DEC return to OG averages within 3 min (10), HSR remains reduced for at least 5 min (10, 12). Importantly, reductions in HSR are particularly evident during covering and recovery runs, close-down/press actions, and running with the ball (12). It was suggested that such impairments may compromise both goal-scoring and defending opportunities by limiting players' ability to reach optimal tactical positions due to fatigue after a WCS (10, 12). Consequently, minimizing reductions in physical performance post-WCS (10) is considered key to optimizing the physical performance itself (1, 9, 13). However, this would require training individualization (8, 14), as both locomotor (4, 5, 15) and mechanical (4) WCS vary depending on playing position, along with contextual factors such as playing status, match half, and total physical output (16).

To train according to individual players’ physical capacities while maintaining tactical and technical specificity, various-sided games (VSG) are commonly used in the training process (3, 17, 18). An important modulator in these games is the relative pitch size per player (19), referred to as the Area per Player (ApP). Based on this, VSG are divided into small-sided games (SSG), medium-sided games (MSG), and large-sided games (LSG), which are defined by an ApP of under 100 m^2^, 100–200 m^2^, and over 200 m^2^, respectively (9). A recent review by Rumpf et al. (19) found that an ApP increase is associated with higher physical output, especially for locomotor variables and, to a lesser extent, mechanical variables. Indeed, an ApP of a minimum of 200 m^2^ was needed to stimulate the 90-min average demands of OG in terms of HSR and SPD (20, 21), while an even higher ApP was suggested to replicate the WCS4 for very-high-speed running (20–24 km·h^−1^, 367 m^2^), SPD (>24 km·h^−1^, 541 m^2^), and TD (245 m^2^) (22). Previous research has shown that neither the locomotor nor the mechanical WCS1 and WCS5 were replicated in LSG when the average values per minute were calculated over the full bout duration (9). Consequently, it has been proposed that identical WCS durations should be compared between LSG and OG (3, 22, 23). For example, Sydney et al. (3) compared the WCS1 of LSG and OG, finding significantly lower values for TD and HSR across all positions during LSG, with an ApP of 240 m^2^ (8 vs. 8) and 270 m^2^ (9 vs. 9). Lacome et al. (23) found that TD and distance covered at speeds greater than 14.4 km·h^−1^ were not significantly different during WCS1, WCS2, WCS3, WCS4, and WCS5 in LSG (10 vs. 10) compared to OG in professional football players, with an ApP (311 m^2^) similar to that of OG. It appears that LSG should incorporate at least an ApP similar to that of official matches in order to replicate WCS, especially for distances covered at higher speeds (>14.4 km·h^−1^).

Although recent studies (3, 23, 24) have compared LSG with the WCS of OG, no study has specifically investigated whether LSG with a comparable ApP can replicate position-specific locomotor and mechanical demands across standardized WCS durations. Therefore, the aim of this study was to investigate whether WCS1, WCS3, and WCS5 of OG can be reproduced during LSG, with an ApP similar to that of OG and with consideration of position-specific demands.

## Materials and methods

### Participants

Twenty-one male football players (21.4 ± 3.00 years of age, 183 ± 4.20 cm, 78.5 ± 6.70 kg, with an average Yo-Yo Intermittent Recovery Test Level 1 of 3,193 m ± 340 m) from the second team of a German football team competing in the second-highest league (2. Bundesliga) participated in this study. The players competed in Germany's fourth division and were classified as Tier 3 according to the participant classification framework (25). The team had five weekly training sessions, with an average weekly training duration of 5:17 ± 0:19 h per week, and one league match. All players followed the team's regular training and match schedule, and only outfield players who were injury-free for the last 6 months and completed 80% of all training sessions were included in the entire (LSG and OG) analysis. Only starting players who completed the entire match were included in the OG analysis. Players were categorized by their playing position as designated by the head coach: CB (*N* = 4), FB (*N* = 4), CM (*N* = 6), WM (*N* = 3), and ST (*N* = 5). Goalkeepers were not included in the study due to their specific requirements. Data collection was conducted routinely during daily training and analyzed a *posteriori*. Consequently, ethics approval was not required (26). Nevertheless, informed written consent was provided by the club and the players. All data were anonymized prior to analysis, and the study was conducted according to the Declaration of Helsinki, local legislation, and institutional requirements.

### Procedures

The study was conducted during the second half of the 2024/25 competitive season, spanning 19 weeks. All sessions were performed on a natural grass surface and conducted at the same time of the day, on match day minus three (MD-3), in alignment with common periodization strategies in football (27, 28). In addition to the 17 OG, two different LSG formats were analyzed. The LSG formats consisted of 10 vs. 10 and 9 vs. 9 formats, with a similar ApP as the OG formats (≈320 m^2^). Each LSG was played for 2–3 bouts with an average bout duration of 15 min (range: 10–22 min) and with 2–5-min of passive recovery between bouts. All games, pitch size, ApP, number of bouts per game, bout, and recovery duration are displayed in Table 1.

**Table 1:** Large-sided-game format with the corresponding pitch size, area per player, the number of bouts, bout duration, and rest time between bouts.

Format	Pitch size	Area per player (m^2^)	Number of bouts	Bout duration (min)	Rest time (min)
9 vs. 9	95 × 68	323	3	15–17	2–5
	95 × 68	323	2	10–12	5
	95 × 67	318	3	10–15	3–5
	95 × 67	318	2	20–21	5
	95 × 67	318	2	15–16	5
	95 × 67	318	3	10–13	3–5
10 vs. 10	105 × 67	320	3	13–15	2–5
	105 × 67	320	2	15–17	5
	105 × 67	320	2	16–22	4
	105 × 67	320	2	20–21	5
	105 × 67	320	4	11–13	3–5
	103 × 68	318	2	15–16	5

There was a total of 698 individual observations (OG = 126, LSG = 570) made. The number of individual observations of each LSG and OG by position can be seen in Table 2.

**Table 2:** Number of individual observations by position during large-sided games (LSG) formats and official games (OG).

Format	CB	FB	CM	WM	ST
9 vs. 9	39	33	55	25	68
10 vs. 10	75	67	118	56	34
LSG total	114	100	173	81	102
OG	29	23	34	14	26

Standard OG rules, including offside, were applied, except that corners, kick-offs, and other interruptions were resumed with an immediate controlled restart. To promote continuous play, spare balls were placed around the field. Verbal encouragement was provided throughout all LSG to maintain a high work rate (29).

### Data collection

External load variables during all training sessions and OG were monitored using 10-Hz Global positioning units (GPS) (Catapult Vector S7, Melbourne, Australia). Each device was activated at least 15 min before data collection to ensure the appropriate acquisition of satellite signals as per the manufacturer's guidelines. Each player wore the same GPS unit to reduce inter-unit differences, as suggested by Riboli et al. (20). The GPS units were positioned in a small pocket located between the players' shoulder blades, integrated into the vest provided by the GPS manufacturer. Following each training session and OG, the GPS data were downloaded and processed using the manufacturer's software (OpenFieldConsole, Version 3.9, Catapult, Melbourne, Australia). The GPS units were found to be reliable and valid (30–32). Excellent inter-system and intra-system reliability were observed for ACC- and DEC-based variables, with an intraclass correlation coefficient (ICC) of 0.91 and 0.97, respectively (31). ICC values from very large to nearly perfect were also reported for locomotor variables, such as maximal sprinting speed (0.97), starting velocity (0.96), and peak power output (0.94). Locomotor and mechanical demands were defined as follows (33): TD was defined as the total distance covered. MSR was defined as the distance covered at a speed of 14.4–19.7 km·h^−1^. HSR was defined as the distance covered at a velocity of 19.8–25.1 km·h^−1^, while SPD was defined as the distance covered at speeds greater than 25.2 km·h^−1^. ACC efforts were classified as low (ACC1, 1.50–2.49 m·s^−2^), moderate (ACC2, 2.50–3.49 m·s^−2^), or high (ACC3, >3.50 m·s^−2^). DEC efforts were defined as low (DEC1, −1.50 to −2.49 m·s^−2^), moderate (DEC2, −2.50 to −3.49 m·s^−2^), or high (DEC3, <−3.50 m·s^−2^). To assess peak positional physical demands, the WCS of OG and LSG were identified using the rolling average method (6, 7) in accordance with recent methodological approaches (3, 23, 34). WCS periods were analyzed for the most common durations, 1 min (WCS1), 3 min (WCS3), and 5 min (WCS5) (2) for both LSG and OG.

### Statistical analysis

All statistical analyses were conducted with R (Version 4.5.0, R Core Team, 2025) using RStudio (Version 2025.02.0 + 496; R Core Team, 2025). Descriptive statistics were reported for all variables (mean ± SD). To examine differences between the playing formats (LSG or OG), in each WCS1, WCS3, or WCS5 period, linear mixed models (LMMs) were employed using the *lme4* package (35). LMMs were chosen due to their robustness in handling unbalanced data sets and repeated measures (35, 36). Each WCS period was analyzed separately, with playing format and player position modeled as fixed effects, while player identity was modeled as a random intercept to account for repeated measures within players. Model assumptions were assessed using visual inspection of Q-Q plots to check for normality, while homoscedasticity was checked using residual vs. fitted values plots. *Post-hoc* comparisons with Bonferroni correction were applied for multiple testing. Effect sizes (ESs) were calculated using Cohen’s *d* and were interpreted as follows (37): trivial (<0.10), small (0.10–0.29), medium (0.30–0.49), large (0.50–0.69), and very large (>0.70). The significance level was set at *p* < 0.05.

## Results

### Locomotor variables

Locomotor variables during WCS1, WCS3, and WCS5 for LSG and OG, stratified by playing position, are presented in Table 3. Across all playing positions and WCS durations, TD, MSR, HSR, and SPD were consistently lower in LSG compared with OG (*p* < 0.05), with ESs ranging from large to very large.

**Table 3:** Total distance (TD), medium-speed-running (MSR), high-speed running (HSR), and sprint distance (SPD) during WCS1, WCS3, and wcs5 for LSG and OG by playing position.

		WCS1		*d*	WCS3		*d*	WCS5		*d*
Position	Variable	LSG	OG		LSG	OG		LSG	OG	
CB	TD	171 ± 15.5**	193 ± 11.6	1.48 (1.04–1.94)	423 ± 37.1**	460 ± 28.0	1.05 (0.62–1.48)	657. ± 61.8**	716 ± 44.0	1.00 (0.57–1.42)
	MSR	52.0 ± 12.9**	74.8 ± 15.1	1.70 (1.24–2.15)	86.5 ± 21.9**	127 ± 24.2	1.83 (1.37–2.30)	117 ± 29.1**	171 ± 36.7	1.73 (1.27–2.19)
	HSR	25.0 ± 11.2**	39.6 ± 10.7	1.31 (0.87–1.74)	35.1 ± 17.7**	51.3 ± 12.1	0.97 (0.54–1.39)	43.8 ± 22.8**	61.9 ± 16.8	0.83 (0.41–1.28)
	SPD	8.74 ± 11.8**	24.6 ± 13.0	1.32 (0.88–1.76)	9.63 ± 13.1**	26.3 ± 14.4	1.24 (0.80–1.67)	10.6 ± 14.4**	27.2 ± 14.2	1.17 (0.73–1.60)
FB	TD	181 ± 17.2**	203 ± 11.9	1.34 (0.85–1.83)	436 ± 38.1**	473 ± 20.9	1.04 (0.56–1.51)	674 ± 62.6**	741 ± 34.1	1.15 (0.67–1.15)
	MSR	56.6 ± 15.6**	82.3 ± 15.1	1.66 (1.16–2.17)	98.6 ± 27.2**	132 ± 15.0	1.33 (0.84–1.82)	133 ± 39.0**	181 ± 29.1	1.27 (0.79–1.76)
	HSR	34.7 ± 14.8**	50.8 ± 11.0	1.14 (0.66–1.62)	51.9 ± 22.4**	73.8 ± 15.0	1.03 (0.57–1.50)	65.5 ± 29.9**	91.8 ± 17.3	0.94 (0.47–1.41)
	SPD	15.5 ± 11.5**	28.9 ± 10.5	1.17 (0.69–1.66)	18.4 ± 14.1**	34.9 ± 12.3	1.20 (0.72–1.69)	20.6 ± 16.7**	38.0 ± 13.5	1.07 (0.80–1.55)
CM	TD	174 ± 16.4**	201 ± 17.7	1.61 (1.21–2.01)	434 ± 43.3**	474 ± 31.5	0.96 (0.58–1.34)	671 ± 74.5**	741 ± 47.2	0.99 (0.61–1.37)
	MSR	55.7 ± 15.6**	81.1 ± 16.7	1.60 (1.20–2.00)	98.01 ± 28.4**	136 ± 28.3	1.32 (0.93–1.71)	133 ± 39.9**	182 ± 37.0	1.26 (0.87–1.65)
	HSR	26.5 ± 11.7**	41.9 ± 13.11	1.28 (0.89–1.67)	36.8 ± 17.0**	61.5 ± 20.5	1.40 (1.01–1.80)	45.5 ± 22.4**	75.1 ± 24.5	1.23 (0.91–1.69)
	SPD	7.75 ± 10.0**	23.9 ± 13.6	1.51 (1.12–1.91)	8.11 ± 10.7**	26.3 ± 14.4	1.60 (1.20–2.00)	8.67 ± 11.6**	29.8 ± 19.2	1.61 (1.21–2.02)
WM	TD	176 ± 15.7**	211 ± 17.1	2.20 (1.54–2.86)	426 ± 37.7**	475 ± 32.0	1.30 (0.70–1.90)	666 ± 56.9**	730 ± 48.5	1.14 (0.54–1.73)
	MSR	52.1 ± 15.1**	77.6 ± 13.1	1.66 (1.04–2.28)	93.3 ± 25.7**	128 ± 15.2	1.44 (0.83–2.05)	127 ± 33.4**	175 ± 21.0	1.51 (0.89–2.12)
	HSR	32.1 ± 11.0**	47.8 ± 12.0	1.40 (0.79–2.00)	48.4 ± 15.6**	73.3 ± 16.7	1.59 (0.97–2.20)	61.9 ± 20.1**	92.9 ± 17.6	1.57 (0.95–2.19)
	SPD	20.4 ± 15.5**	39.7 ± 12.1	1.28 (0.67–1.88)	24.1 ± 18.4**	47.8 ± 15.8	1.31 (0.71–1.92)	28.6 ± 22.5**	56.9 ± 20.8	1.27 (0.66–1.87)
ST	TD	178 ± 14.7**	202 ± 16.9	1.58 (1.10–2.06)	433 ± 36.8**	469 ± 32.1	1.00 (0.55–1.45)	669 ± 59.5**	726 ± 44.1	1.00 (0.55–1.45)
	MSR	53.3 ± 12.4**	75.1 ± 12.7	1.76 (1.27–2.24)	95.2 ± 22.8**	126 ± 24.0	1.29 (0.83–1.75)	128 ± 31.0**	175 ± 21.0	1.33 (0.86–1.79)
	HSR	32.1 ± 10.9**	48.1 ± 10.6	1.47 (1.00–1.94)	46.9 ± 15.9**	71.5 ± 14.0	1.58 (1.10–2.06)	58.9 ± 21.7**	88.6 ± 18.2	1.41 (0.94–1.88)
	SPD	13.1 ± 11.7**	29.2 ± 9.81	1.41 (0.94–1.88)	14.2 ± 13.1**	36.3 ± 16.3	1.60 (1.12–2.07)	16.3 ± 15.9**	40.4 ± 20.9	1.41 (0.94–1.88)

Values are presented as mean ± SD. *d* = Cohen's *d* effect size.

* *p* < 0.05.

** *p* < 0.001.

### Mechanical variables

Mechanical variables during WCS1, WCS3, and WCS5 for LSG and OG, stratified by playing position, are presented in Table 4.

**Table 4:** Low-, medium- and high intensity accelerations (ACC1, ACC2, and ACC3) and decelerations (DEC1, DEC2, and DEC3) during WCS1, WCS3, and WCS5 for LSG and OG by playing position.

		WCS1		*p*	*d*	WCS3		*p*	*d*	WCS5		*p*	*d*
Position	Variable	LSG	OG			LSG	OG			LSG	OG		
CB	ACC1	5.40 ± 1.17**	6.31 ± 1.47	<0.001	0.73 (0.31–1.15)	10.1 ± 2.29**	11.6 ± 2.58	<0.001	0.64 (0.22–1.06)	14.0 ± 3.28**	16.1 ± 3.63	<0.001	0.63 (0.22–1.05)
	ACC2	2.43 ± 0.75**	3.24 ± 0.74	<0.001	1.08 (0.65–1.52)	3.76 ± 1.31**	5.14 ± 1.51	<0.001	1.02 (0.58–1.44)	4.87 ± 1.79**	6.52 ± 1.99	<0.001	0.90 (0.48–1.33)
	ACC3	0.71 ± 0.61**	1.34 ± 0.61	<0.001	1.04 (0.62–1.47)	0.83 ± 0.80**	1.66 ± 0.72	<0.001	1.05 (0.62–1.48)	0.92 ± 0.90**	1.90 ± 0.90	<0.001	1.08 (0.65–1.51)
	DEC1	4.58 ± 1.06**	5.59 ± 1.21	<0.001	0.92 (0.50–1.35)	8.35 ± 2.07**	9.93 ± 2.39	<0.001	0.74 (0.32–1.16)	11.2 ± 2.84**	13.3 ± 3.28	<0.001	0.71 (0.29–1.13)
	DEC2	2.02 ± 0.85**	3.03 ± 0.73	<0.001	1.23 (0.79–1.66)	3.27 ± 1.47**	4.24 ± 1.18	<0.001	0.69 (0.27–1.10)	4.15 ± 1.89**	5.41 ± 1.38	<0.001	0.70 (0.28–1.12)
	DEC3	1.16 ± 0.72**	1.76 ± 0.58	<0.001	0.86 (0.44–1.29)	1.50 ± 1.01**	2.41 ± 0.78	<0.001	0.95 (0.52–1.37)	1.78 ± 1.21**	2.97 ± 0.94	<0.001	1.01 (0.59–1.45)
FB	ACC1	5.20 ± 1.33*	5.91 ± 1.44	0.036	0.53 (0.07–0.99)	9.57 ± 2.60	10.3 ± 2.31	0.397	0.30 (−0.15 to 0.76)	13.3 ± 3.63	14.1 ± 3.60	0.640	0.24 (−0.22 to 0.70)
	ACC2	2.58 ± 0.81**	3.26 ± 0.69	<0.001	0.87 (0.40–1.34)	4.10 ± 1.42*	5.26 ± 1.45	0.002	0.82 (0.35–1.28)	5.29 ± 1.87*	6.74 ± 2.14	0.005	0.75 (0.29–1.22)
	ACC3	1.17 ± 0.53	1.39 ± 0.66	0.235	0.40 (0.06–0.86)	1.57 ± 0.88	1.87 ± 1.01	0.231	0.33 (−0.13 to 0.79)	1.76 ± 1.01	2.17 ± 1.37	0.153	0.38 (−0.08 to 0.84)
	DEC1	4.77 ± 1.16*	5.52 ± 0.99	0.014	0.66 (0.20–1.13)	8.43 ± 2.27	9.65 ± 2.04	0.002	0.55 (0.09–1.01)	11.2 ± 3.18*	13.0 ± 2.93	0.043	0.58 (0.12–1.05)
	DEC2	2.51 ± 0.93**	3.30 ± 0.76	<0.001	0.88 (0.41–1.35)	3.94 ± 1.44**	5.09 ± 1.35	<0.001	0.80 (0.34–1.27)	5.04 ± 2.00**	6.74 ± 1.79	<0.001	0.87 (0.39–1.34)
	DEC3	1.52 ± 0.64**	2.35 ± 0.78	<0.001	1.24 (0.75–1.72)	2.14 ± 1.03**	3.30 ± 0.97	<0.001	1.15 (0.67–1.63)	2.51 ± 1.21**	4.17 ± 1.23	<0.001	1.37 (0.88–1.86)
CM	ACC1	5.00 ± 1.14**	5.91 ± 1.16	<0.001	0.80 (0.42–1.17)	9.23 ± 2.17*	10.1 ± 2.01	0.009	0.40 (0.03–0.77)	12.7 ± 3.07*	13.8 ± 2.65	0.012	0.36 (0.01–0.73)
	ACC2	2.42 ± 0.92**	2.82 ± 0.67	<0.001	0.45 (0.08–0.83)	3.85 ± 1.65	4.12 ± 1.01	0.080	0.17 (−0.19 to 0.54)	4.90 ± 2.14*	5.50 ± 1.62	0.015	0.29 (0.07–0.66)
	ACC3	0.75 ± 0.63**	1.21 ± 0.59	<0.001	0.74 (0.35–1.11)	0.95 ± 0.90**	1.47 ± 0.75	<0.001	0.60 (0.22–0.97)	1.04 ± 1.02**	1.82 ± 0.94	<0.001	0.78 (0.40–1.15)
	DEC1	4.72 ± 1.23**	5.68 ± 1.04	<0.001	0.80 (0.42–1.18)	8.48 ± 2.21**	10.3 ± 1.89	<0.001	0.85 (0.48–1.23)	11.6 ± 3.09**	14.2 ± 2.38	<0.001	0.85 (0.47–1.23)
	DEC2	2.30 ± 0.79**	3.24 ± 0.78	<0.001	1.19 (0.80–1.57)	3.57 ± 1.37**	4.85 ± 0.99	<0.001	0.98 (0.60–1.36)	4.56 ± 1.78**	6.21 ± 1.27	<0.001	0.96 (0.58–1.35)
	DEC3	1.14 ± 0.62**	1.79 ± 0.64	<0.001	1.05 (0.66–1.43)	1.49 ± 0.89**	2.62 ± 0.92	<0.001	1.26 (0.87–1.65)	1.66 ± 1.01**	2.97 ± 1.00	<0.001	1.30 (0.91–1.69)
WM	ACC1	4.89 ± 1.08*	5.86 ± 1.10	0.006	0.89 (0.30–1.48)	8.65 ± 1.84*	10.1 ± 1.96	0.024	0.80 (0.22–1.38)	11.9 ± 2.60*	14.3 ± 2.40	0.012	0.90 (0.31–1.49)
	ACC2	2.67 ± 1.00**	3.93 ± 0.83	<0.001	1.29 (0.69–1.89)	4.48 ± 1.70*	5.57 ± 1.40	0.006	0.65 (0.07–1.24)	5.85 ± 2.32**	7.57 ± 1.83	<0.001	0.76 (0.18–1.35)
	ACC3	1.20 ± 0.68**	2.00 ± 0.55	<0.001	1.21 (0.61–1.81)	1.64 ± 1.03**	2.57 ± 0.85	<0.001	0.92 (0.33–1.51)	1.95 ± 1.26**	3.14 ± 0.77	<0.001	0.99 (0.40–1.58)
	DEC1	4.21 ± 1.11**	5.79 ± 1.05	<0.001	1.42 (0.81–2.03)	7.59 ± 1.93**	9.86 ± 2.25	<0.001	1.15 (0.55–1.74)	10.2 ± 2.65**	13.1 ± 2.67	<0.001	1.08 (0.48–1.67)
	DEC2	2.75 ± 0.84*	3.43 ± 0.51	0.004	0.84 (0.25–1.42)	4.52 ± 1.44*	5.36 ± 0.84	0.032	0.61 (0.03–1.19)	5.85 ± 1.98**	6.79 ± 0.97	<0.001	0.50 (−0.08 to 1.08)
	DEC3	1.98 ± 0.63**	2.71 ± 0.83	<0.001	1.12 (0.52–1.71)	2.91 ± 0.91**	3.93 ± 1.07	<0.001	1.09 (0.49–1.68)	3.56 ± 1.14**	5.29 ± 1.59	<0.001	1.43 (0.82–2.04)
ST	ACC1	5.07 ± 1.13*	5.69 ± 1.05	0.011	0.56 (0.12–1.00)	9.06 ± 2.06	9.50 ± 1.53	0.258	0.22 (−0.21 to 0.66)	12.6 ± 3.08	13.0 ± 1.79	0.409	0.14 (−0.29 to 0.58)
	ACC2	2.22 ± 0.80**	3.35 ± 0.75	<0.001	1.43 (0.96–1.89)	3.44 ± 1.27**	5.23 ± 1.07	<0.001	1.45 (0.98–1.92)	4.46 ± 1.73**	6.92 ± 1.62	<0.001	1.44 (0.97–1.91)
	ACC3	0.95 ± 0.69*	1.03 ± 0.73	0.005	0.59 (0.15–1.03)	1.20 ± 0.93**	1.85 ± 0.83	<0.001	0.71 (0.27–1.15)	1.33 ± 1.09**	2.23 ± 0.91	<0.001	0.85 (0.40–1.29)
	DEC1	4.61 ± 1.09*	5.12 ± 0.82	0.023	0.48 (0.05–0.92)	8.23 ± 1.89	8.58 ± 1.53	0.286	0.19 (−0.24 to 0.63)	11.2 ± 2.72	12.0 ± 2.29	0.103	0.31 (−0.13 to 0.75)
	DEC2	2.64 ± 0.82*	3.08 ± 0.69	0.003	0.55 (0.11–0.99)	4.14 ± 1.52**	5.00 ± 1.17	<0.001	0.59 (0.15–1.03)	5.41 ± 1.91**	6.65 ± 1.62	<0.001	0.67 (0.23–1.11)
	DEC3	1.44 ± 0.65**	2.23 ± 0.51	<0.001	1.26 (0.79–1.72)	2.07 ± 1.05**	3.27 ± 0.92	<0.001	1.17 (0.72–1.63)	2.56 ± 1.38**	4.15 ± 1.12	<0.001	1.19 (0.74–1.65)

Values are presented as mean ± SD. *d* = Cohen's *d* effect size.

* *p* < 0.05.

** *p* < 0.001.

ACC1 values were significantly lower in LSG compared to OG across all positions during WCS1 (large to very large effects), while during WCS3 and WCS5, significant reductions were only observed in CB, CM, and WM with very large ESs. Similarly, ACC2 was significantly lower in LSG compared to OG for all positions in WCS1 (medium to very large effect sizes) and WCS5 (small to very large effect sizes). During WCS3, only CB, ST, WM, and FB (large to very large effect sizes) displayed significantly lower values. During WCS1, WCS3, and WCS5, all positions except for FB displayed significantly lower ACC3 values with large to very large ESs.

DEC1 was significantly lower in LSG than in OG across all positions during WCS1 (medium to very large effect sizes). During WCS3 and WCS5, only CM, CB, and WM (large to very large effect sizes) experienced significantly lower values during LSG than OG, while for FB, similar reductions were found only during WCS5. Both DEC2 and DEC3 were significantly lower in LSG compared to OG for all positions in WCS1, WCS3, and WCS5, with ESs ranging from large to very large.

## Discussion

This study aimed to investigate whether the positional, locomotor, and mechanical demands during WCS1, WCS3, and WCS5 were replicated within LSG with an ApP similar to that of OG. For all positions and WCS durations, the locomotor variables (TD, MSR, HSR, and SPD) of WCS were significantly lower in LSG compared to OG, indicating a substantial underrepresentation of locomotor WCS demands in LSG. These results are in line with those of Riboli and Castagna (14), who found significantly lower values for all locomotor variables in WCS1, WCS2, WCS3, WCS4, and WCS5 for SSG. However, readers need to be cognizant of the large variability in ApP (38–340 m^2^) utilized in the games investigated by Riboli and Castagna (14). Conversely, Riboli et al. (22) suggested that the TD during WCS4 could be replicated using a minimum ApP of 246 m^2^. Furthermore, Sydney et al. (3) indicated that during WCS1, an even higher ApP might be needed, as significantly lower TD values were found when using ApPs of 240 and 270 m^2^ in youth footballers. The current study used an even higher ApP (≈320 m^2^), but still failed to reproduce the TD and the MSR of WCS1, WCS3, and WCS5 during OG. Lacome et al. (23) reported that TD and distance covered above 14.4 km·h^−1^ during WCS1- WCS5 were successfully replicated in LSG performed by professional football players when a comparable ApP was implemented. Nevertheless, when the broader body of evidence is considered, the capacity of ApP in LSG to consistently reproduce OG WCS demands for TD and MSR remain inconclusive.

Higher speed thresholds, such as HSR, are particularly important as post-WCS reductions during OG may compromise goal-scoring and defending opportunities (12), emphasizing the need to adequately prepare players for these demands while supporting injury prevention (38). Thus, HSR (>19.8 km·h^−1^) was investigated by Sydney et al. (3), who found significantly lower values in LSG compared to OG in youth football players, using an ApP of 240 and 270 m^2^. Expanding on the above, this study did not replicate WCS1, WCS3, or WCS5 for HSR or SPD, even when using a larger ApP (≈320 m^2^) than Sydney et al. (3). While it is well documented (9, 19–21, 39) that an increase in ApP leads to an increase in locomotor output, an ApP similar to OG (320 m²) appears insufficient to replicate the HSR and SPD demands of WCS1, WCS3, and WCS5 of OG. This aligns with the findings of Riboli et al. (22), who found that only an ApP larger than OG (>320 m²) could replicate the WCS4 of running at high speeds (20–24 km·h^−1^) and SPD (>24 km·h^−1^). It should be acknowledged that contextual factors during training typically involve lower competitive pressure and reduced motivational and psychological demands (9). In addition, pacing strategies (40–42) and fewer transitional phases (43) may influence how WCS emerge during LSG compared to OG and may therefore contribute to lower exercise intensity during training sessions.

Given the current inconsistent findings across the literature for TD and MSR and the consistently lower values at higher speed thresholds (HSR, SPD) in LSG compared to OG, coaches should choose training drills that have proven effective at replicating WCS. While Riboli and Castagna (14) suggested that an ApP larger than OG (341–640 m^2^) may replicate WCS1—WCS5 locomotor demands <24 km·h^−1^, this approach may reduce technical-tactical specificity. From a practical perspective, substantially increasing the ApP above that of the OG could compromise representativeness and may not easily transfer to applied training contexts. The same study (14) identified running-based drills as an effective approach to achieving higher values for TD and HSR (15–20 km·h^−1^), but not for higher speed thresholds (>20 km·h^−1^). As multiple running-based drills were analyzed together and specific protocols were not mentioned, it is impossible to determine the training protocol. Notably, for the 30-s WCS, running-based drills displayed significantly higher HSR and SPD values, with runs covering 75 meters in 12–15 s and similar values observed during 50-m shuttles in 7–10 s (8). Therefore, running drills appear to be a promising method for targeting WCS, but they should still be prescribed according to position-specific OG demands. In the present study, WM had to cover 39.7, 47.8, 77.6, and 211 meters for SPD, HSR, MSR, and TD, respectively, during WCS1. From a practical perspective, replicating WCS1 demands may therefore require slightly longer running distances to account for acceleration (44). For example, a training drill could include one 50-m sprint (<9 s, SPD), one 56-m run (9–11 s, HSR), and one 80-m run (<20 s, MSR) with 10 m (10 s) of active recovery (i.e., walking or low-intensity jogging) between each effort.

In terms of mechanical demands, ACC1, ACC2, and ACC3 were significantly lower in WCS1 during LSG for all positions, except in FB in ACC3. ACC1 loads were similar in LSG compared to OG in ST (WCS3 and WCS5) and FB (WCS3 and WCS5), while ACC2 loads were similar only in CM (WCS3). For ACC3, only FB experienced an identical external load in LSG compared to OG in WCS3. These findings partly extend the previous work by de Dios Alvarez et al. (9), who reported that ACC (>3 m·s^−2^) during WCS1 and WCS5 was significantly lower in LSG compared to OG for all positions. While the study by de Dios Alvarez et al. (9) was conducted with youth football players, the current study displayed similar results and suggests that LSG may not be the optimal format for replicating ACC WCS demands. In contrast, SSG appear to be a more suitable format for replicating ACC demands, as those were shown to display significantly higher ACC (>3 m·s^−2^) values than OG WCS5 (9, 34). Also, MSG displayed similar WCS5 ACC (>2 m·s^−2^) values in a study by Dalen et al. (45), while significantly higher (≈150%) values for ACC WCS5 demands (>3 m·s^−2^) were found by Martin-Garcia et al. (34) Additionally, MSG also replicated the 30-s WCS demands for ACC (>3 m·s^−2^) in a study by Bortnik et al. (8) The authors found similar values in a 10 vs. 10 format and even significantly higher values in formats with fewer players (4 vs. 4 and 5 vs. 5) (8). Unlike LSG, SSG, and MSG, who displayed similar or even higher values than OG WCS, possibly due to an inverse relationship between speed and acceleration intensity, meaning that those players often start from lower velocities, allowing for higher acceleration peaks (33).

DEC metrics were significantly lower in LSG compared to OG across all thresholds and positions. The only exceptions were ST (WCS3 and WCS5) and FB (WCS3) for DEC1. Consistent with the findings for ACC values, DEC (<−3 m·s^−2^) values during the WCS of 30 s in MSG were investigated by Bortnik et al. (8), finding similar values in 10 vs. 10 and significantly higher values in formats with fewer players (4 vs. 4, 5 vs. 5) compared to OG. During WCS5 Martin-Garcia et al. (34) stated that ≈150% of OG DEC values were obtained during MSG and during SSG. Coaches looking to replicate ACC and DEC should use SSG and MSG formats, as they have been shown to be effective in reproducing WCS demands (8, 34, 45). Moreover, SSG and MSG with fewer players (i.e., 4 vs. 4 or 5 vs. 5) appear to be even more suitable, as they produced even higher ACC and DEC values (8, 34, 45). This could explain the discrepancies in WCS for ACC and DEC during LSG compared to OG in this study.

While this study provides valuable insights for practitioners seeking to replicate WCS during football practice, some limitations should be acknowledged. First, different thresholds for locomotor and mechanical WCS in different studies may make comparisons difficult. However, for HSR, SPD, ACC, and DEC variables, results remained consistent regardless of the thresholds (9, 34, 46). Second, only LSG formats of 9 vs. 9 and 10 vs. 10 were examined. Given that a reduction in the number of players may result in greater exercise intensity (19), LSG with a sufficiently large ApP (i.e., 320 m^2^) and with a smaller number of players (i.e., 5 vs. 5) could possibly prove sufficient in replicating WCS for all locomotor and mechanical variables. Future research should explore whether such formats can replicate external load metrics while maintaining the technical-tactical aspects of OG. Finally, this investigation was limited to a single team, conducted with the second squad of a professional football team; therefore, relatively small positional subgroups may limit generalizability. To make these findings more generalizable, further studies should replicate the design across multiple age groups and competitive levels, incorporating larger sample sizes.

## Conclusion

This study demonstrated that 9 vs. 9 and 10 vs. 10 LSG, with an ApP similar to OG, may be insufficient to replicate the locomotor demands of OG in WCS1, WCS3, and WCS5 in professional football players. Similarly, LSG generally failed to replicate the mechanical OG demands during WCS1, WCS3, and WCS5, with some exceptions depending on WCS duration and player position. However, these exceptions should be interpreted cautiously due to the variability rather than true physiological differences.

## Data Availability

The data analyzed in this study is subject to the following licenses/restrictions: data of the findings of the study is available on reasonable request from the corresponding author. Requests to access these datasets should be directed to Lukas Herzig, sr.lukas-herzig@gmx.de.

## References

[1] Douchet T Michel A Verdier J Babault N Gosset M Delaval B. Intensity vs. volume in professional soccer: comparing congested and non-congested periods in competitive and training contexts using worst-case scenarios. Sports. (2025) 13:70. 10.3390/sports13030070 40137794 PMC11946561

[2] Rico-González M Oliveira R Palucci Vieira LH Pino-Ortega J Clemente FM. Players’ performance during worst-case scenarios in professional soccer matches: a systematic review. Biol Sport. (2022) 39:695–713. 10.5114/biolsport.2022.107022 35959320 PMC9331336

[3] Sydney MG Wollin M Chapman DW Ball N Mara JK. Do conditioning focused various-sided training games prepare elite youth male soccer players for the demands of competition? Biol Sport. (2022) 39:825–32. 10.5114/biolsport.2022.109454 36247949 PMC9536390

[4] Lobo-Triviño D García-Calvo T Polo-Tejada J Sanabria-Pino B López del Campo R Nevado-Garrosa F Analyzing positional and temporal variations in worst-case scenario demands in professional Spanish soccer. J Funct Morphol Kinesiol. (2025) 10:172. 10.3390/jfmk10020172 40407456 PMC12101334

[5] Oliva-Lozano JM Rojas-Valverde D Gómez-Carmona CD Fortes V Pino-Ortega J. Worst case scenario match analysis and contextual variables in professional soccer players: a longitudinal study. Biol Sport. (2020) 37:429–36. 10.5114/biolsport.2020.97067 33343077 PMC7725043

[6] Cunningham DJ Shearer DA Carter N Drawer S Pollard B Bennett M Assessing worst case scenarios in movement demands derived from global positioning systems during international rugby union matches: rolling averages versus fixed length epochs. PLoS One. (2018) 13:e0195197. 10.1371/journal.pone.0195197 29621279 PMC5886488

[7] Doncaster G Page R White P Svenson R Twist C. Analysis of physical demands during youth soccer match-play: considerations of sampling method and epoch length. Res Q Exerc Sport. (2020) 91:326–34. 10.1080/02701367.2019.1669766 31774386

[8] Bortnik L Nir O Forbes N Alexander J Harper D Bruce-Low S Worst case scenarios in soccer training and competition: analysis of playing position, congested periods, and substitutes. Res Q Exerc Sport. (2024) 95:588–600. 10.1080/02701367.2023.2290265 38100605

[9] de Dios-Álvarez V Castellano J Padrón-Cabo A Rey E. Do small-sided games prepare players for the worst-case scenarios of match play in elite young soccer players? Biol Sport. (2023) 41:95–106. 10.5114/biolsport.2024.127389 PMC10765451 38188112

[10] Schimpchen J Gopaladesikan S Meyer T. The intermittent nature of player physical output in professional football matches: an analysis of sequences of peak intensity and associated fatigue responses. Euro J Sport Sci. (2021) 21:793–802. 10.1080/17461391.2020.1776400 32466742

[11] Fang Z Wang Z Li X Gómez M-A Liu H. Physical and technical performance in and after the worst-case scenario in matches of the Chinese super league of soccer. Biol Sport. (2024) 42:95–103. 10.5114/biolsport.2025.142642 40182710 PMC11963117

[12] Ju W Doran D Hawkins R Gómez-Díaz A Martin-Garcia A Ade JD Contextualised peak periods of play in English premier league matches. Biol Sport. (2022) 39:973–83. 10.5114/biolsport.2022.112083 36247964 PMC9536363

[13] Díez A Bataller-Cervero AV Mainer-Pardos E Roso-Moliner A Arjol-Serrano JL Lozano D. Comparison of the worst-case scenarios between training and competition weeks for each playing position in an elite football season. Biol Sport. (2025) 42:135–44. 10.5114/biolsport.2025.148538 PMC12490310 41048233

[14] Riboli A Castagna C. Soccer-drill specificity in top-class male players with reference to peak match locomotor demands. J Sports Sci. (2023) 41:573–83. 10.1080/02640414.2023.2228131 37343952

[15] Martín-García A Casamichana D Díaz AG Cos F Gabbett TJ. Positional differences in the most demanding passages of play in football competition. J Sports Sci Med. (2018) 17:563–70. 30479524 PMC6243617

[16] Novak AR Impellizzeri FM Trivedi A Coutts AJ McCall A. Analysis of the worst-case scenarios in an elite football team: towards a better understanding and application. J Sport Sci. (2021) 39:1850–9. 10.1080/02640414.2021.1902138 33840362

[17] Hill-Haas SV Dawson B Impellizzeri FM Coutts AJ. Physiology of small-sided games training in football: a systematic review. Sports Med. (2011) 41:199–220. 10.2165/11539740-000000000-00000 21395363

[18] Chena M Morcillo-Losa JA Rodríguez-Hernández ML Asín-Izquierdo I Pastora-Linares B Zapardiel JC. Workloads of different soccer-specific drills in professional players. J Hum Kin. (2022) 84:135–47. 10.2478/hukin-2022-000075 PMC9679172 36457458

[19] Rumpf M Jäger J Clemente F Altmann S Lochmann M. The effect of relative pitch size on physiological, physical, technical and tactical variables in small-sided games: a literature review and practical guide. Front Sports Act Living. (2025) 7:592536. 10.3389/fspor.2025.1592536 PMC12089100 40395810

[20] Riboli A Coratella G Rampichini S Cé E Esposito F. Area per player in small-sided games to replicate the external load and estimated physiological match demands in elite soccer players. PLoS One. (2020) 15:e0229194. 10.1371/journal.pone.0229194 32966305 PMC7510966

[21] Riboli A Esposito F Coratella G. Technical and locomotor demands in elite soccer: manipulating area per player during small-sided games to replicate official match demands. Biol Sport. (2023) 40:639–47. 10.5114/biolsport.2023.118338 37398955 PMC10286612

[22] Riboli A Esposito F Coratella G. Small-sided games in elite football: practical solutions to replicate the 4-min match-derived maximal intensities. J Strength Cond Res. (2023) 37:366–74. 10.1519/JSC.0000000000004249 35333202

[23] Lacome M Simpson BM Cholley Y Lambert P Buchheit M. Small-sided games in elite soccer: does one size fit all? Int J Sports Physiol Perform. (2018) 13:568–76. 10.1123/ijspp.2017-0214 28714774

[24] Casamichana D Ulloa I Agirrezabalaga O Etxeazarra I González de Suso JM Azurza A Can we replicate the most demanding periods of official football matches in large-sided training games? J Funct Morphol Kinesiol. (2025) 10:410. 10.3390/jfmk10040410 41133600 PMC12550972

[25] McKay AKA Stellingwerff T Smith ES Martin DT Mujika I Goosey-Tolfrey VL Defining training and performance caliber: a participant classification framework. Int J Physiol Perform. (2021) 17:317–31. 10.1123/ijspp.2021-0451 34965513

[26] Winter EM Maughan RJ. Requirements for ethics approvals. J Sports Sci. (2009) 27:985. 10.1080/02640410903178344 19847681

[27] Buchheit M Sandua M Berndsen J Shelton A Smith S Norman D Loading patterns and programming practices in elite football: insights from 100 elite practitioners. Sport Perfrom Sci Rep. (2021) 1(153):1–18.

[28] Morgans R Rhodes D Teixeira J Modric T Versic S Oliveira R. Quantification of training load across two competitive seasons in elite senior and youth male soccer players from an English premiership club. Biol Sport. (2023) 40:1197–205. 10.5114/biolsport.2023.126667 37867738 PMC10588577

[29] de Dios-Álvarez V Padrón-Cabo A Alkain-Villa P Rey E Castellano J. Area per player in small-sided games to estimate the external load in elite youth soccer players. J Hum Kinet. (2024) 95:123–38. 10.5114/jhk/189421 39944978 PMC11812169

[30] Crang ZL Duthie G Cole MH Weakley J Hewitt A Johnston RD. The validity of raw custom-processed global navigation satellite systems data during straight-line sprinting across multiple days. J Sci Med Sport. (2024) 27:204–10. 10.1016/j.jsams.2023.12.004 38195366

[31] Mackay L Sawczuk T Jones B Darrall-Jones J Clark A Whitehead S. The reliability of a commonly used (CatapultTM vector S7) microtechnology unit to detect movement characteristics used in court-based sports. J Sports Sci. (2025) 43:555–64. 10.1080/02640414.2025.2468585 39994076

[32] Cormier P Tsai MC Meylan C Agar-Newman D Epp-Stobbe A Kalthoff Z Concurrent validity and reliability of different technologies for sprint-derived horizontal force-velocity-power profiling. J Strength Cond Res. (2023) 37:1298–305. 10.1519/JSC.0000000000004429 36727987

[33] Cotteret C González-de-la-Flor Á Prieto Bermejo J Almazán Polo J Jiménez Saiz SL. A narrative review of the velocity and acceleration profile in football: the influence of playing position. Sports. (2025) 13:18. 10.3390/sports13010018 39852614 PMC11769499

[34] Martin-Garcia A Castellano J Diaz AG Cos F Casamichana D. Positional demands for various-sided games with goalkeepers according to the most demanding passages of match play in football. Biol Sport. (2019) 36:171–80. 10.5114/biolsport.2019.83507 31223195 PMC6561222

[35] Bates D Mächler M Bolker B Walker S. Fitting linear mixed-effects models using lme4. J Stat Softw. (2015) 67:1–48. 10.18637/jss.v067.i01

[36] Winter B. Linear models and linear mixed effects models in R with linguistic applications. (2013). 10.48550/arXiv.1308.5499

[37] Cohen J. Statistical Power Analysis for the Behavioral Sciences. 2nd ed. Hillsdale, NJ: Lawrence Erlbaum Associates (1988). p. 1.

[38] Gómez-Piqueras P Alcaraz PE. If you want to prevent hamstring injuries in soccer, run fast: a narrative review about practical considerations of sprint training. Sports. (2024) 12:134. 10.3390/sports12050134 38787003 PMC11126098

[39] Riboli A Olthof SBH Esposito F Coratella G. Training elite youth soccer players: area per player in small-sided games to replicate the match demands. Biol Sport. (2021) 39:579–98. 10.5114/biolsport.2022.106388 35959338 PMC9331353

[40] Köklü Y Alemdaroğlu U Cihan H Wong DP. Effects of bout duration on players’ internal and external loads during small-sided games in young soccer players. Int J Sports Physiol Perform. (2017) 12:1370–7. 10.1123/ijspp.2016-0584 28338366

[41] Gabbett TJ Walker B Walker S. Influence of prior knowledge of exercise duration on pacing strategies during game-based activities. Int J Sports Physiol Perform. (2015) 10:298–304. 10.1123/ijspp.2013-0543 25158210

[42] Sampson JA Fullagar HHK Gabbett T. Knowledge of bout duration influences pacing strategies during small-sided games. J Sports Sci. (2015) 33:85–98. 10.1080/02640414.2014.925571 24904981

[43] Olthof SBH Frencken WGP Lemmink KAPM. When something is at stake: differences in soccer performance in 11 vs. 11 during official matches and training games. J Strength Cond Res. (2019) 33:167–73. 10.1519/JSC.0000000000002936 30566410 PMC6314497

[44] Pimenta R Maia F Silva H Nakamura FY. The speed dynamics of different sprint and acceleration exercises applied during football training. Sci Rep. (2025) 15:29543. 10.1038/s41598-025-04641-w 40796906 PMC12343760

[45] Dalen T Sandmæl S Stevens TGA Hjelde GH Kjøsnes TN Wisløff U. Differences in acceleration and high-intensity activities between small-sided games and peak periods of official matches in elite soccer players. J Strength Cond Res. (2021) 35:2018. 10.1519/JSC.0000000000003081 30741867

[46] Toivonen RM Rantalainen T Walker S. The impact of varying small-sided games’ pitch sizes on replicating official match physical demands during effective playing time in male professional soccer players. Int J Perform Anal Sport. (2025) 1–18. 10.1080/24748668.2025.2490867

